# Protection against H_2_O_2_-evoked toxicity in HT22 hippocampal neuronal cells by geissoschizine methyl ether via inhibiting ERK pathway

**DOI:** 10.1515/tnsci-2022-0243

**Published:** 2022-10-10

**Authors:** Shengquan Hu, Lei Yang, Yucui Ma, Limin Li, Zhiyue Li, Xiaomin Wen, Zhengzhi Wu

**Affiliations:** Shenzhen Institute of Translational Medicine/Shenzhen Institute of Geriatrics, The First Affiliated Hospital of Shenzhen University, Shenzhen Second People’s Hospital, Shenzhen, Guangdong Province, China; Department of Spine Surgery, The First Affiliated Hospital of Shenzhen University, Shenzhen Second People’s Hospital, Shenzhen, Guangdong Province, China; School of Chinese Medicine, Southern Medical University, Guangzhou, Guangdong Province, China

**Keywords:** neurodegenerative diseases, geissoschizine methyl ether, neuroprotection, ERK pathway, p38 pathway

## Abstract

Oxidative stress is considered as an important mechanism underlying the pathology of neurodegenerative disorders. In this study, we utilized an *in vitro* model where oxidative stress process was evoked by exogenous hydrogen peroxide (H_2_O_2_) in HT22 murine hippocampal neurons and evaluated the neuroprotective effects of geissoschizine methyl ether (GME), a naturally occurring alkaloid from the hooks of *Uncaria rhynchophylla* (Miq.) Jacks. After a 24 h H_2_O_2_ (350 μM) insult, a significant decrease in cell survival and a sharp increase in intracellular reactive oxygen species were observed in HT22 cells. Encouragingly, GME (10–200 μM) effectively reversed these abnormal cellular changes induced by H_2_O_2_. Moreover, mechanistic studies using Western blot revealed that GME inhibited the increase of phospho-ERK protein expression, but not phospho-p38, caused by H_2_O_2_. Molecular docking simulation further revealed a possible binding mode that GME inhibited ERK protein, showing that GME favorably bound to ERK via multiple hydrophobic and hydrogen bond interactions. These findings indicate that GME provide effective neuroprotection via inhibiting ERK pathway and also encourage further *ex vivo* and *in vivo* pharmacological investigations of GME in treating oxidative stress-mediated neurological disorders.

## Graphical abstract

**Figure:**
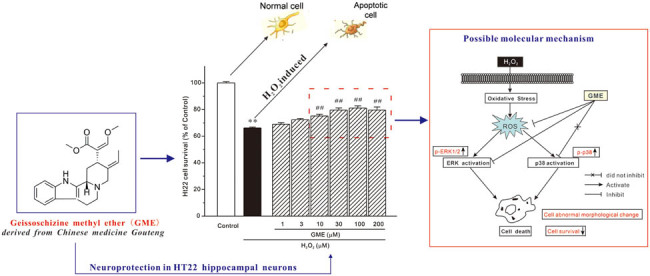

## 1 Introduction

Oxidative stress is widely accepted as a critical mechanism that underlies the pathology of neurological disorders, the best known of which are Alzheimer’s and Parkinson’s conditions [1,2]. Excess reactive oxygen species (ROS), over-generated during oxidative stress process, results in progressive neuronal death in a variety of brain regions particularly hippocampus, cortex, and substantia nigra [3,4], as these areas have relatively larger amount of active oxygen but lower levels of antioxidant enzymes. Hydrogen peroxide (H_2_O_2_) generates superoxide and hydroxyl radicals, the major components of ROS, and has thus been widely employed to mimic oxidative stress in different cellular paradigms including HT22 murine hippocampal neurons and differentiated PC12 cells [5–7]. It is believed that H_2_O_2_-mediated oxidative stress in neuronal cells triggers possible molecular mechanisms that, through the over-stimulation of mitogen-activated protein kinases (MAPKs) [8,9] and/or glycogen synthase kinase-3β (GSK3β) [10,11], lead to cell injury. Since there is an imbalance between the generation and clearance of ROS under the pathophysiology of neurodegenerative conditions, small molecules that could protect H_2_O_2_-induced toxicity may have therapeutic significance in treating these devastating diseases [12].

**Figure 1:**
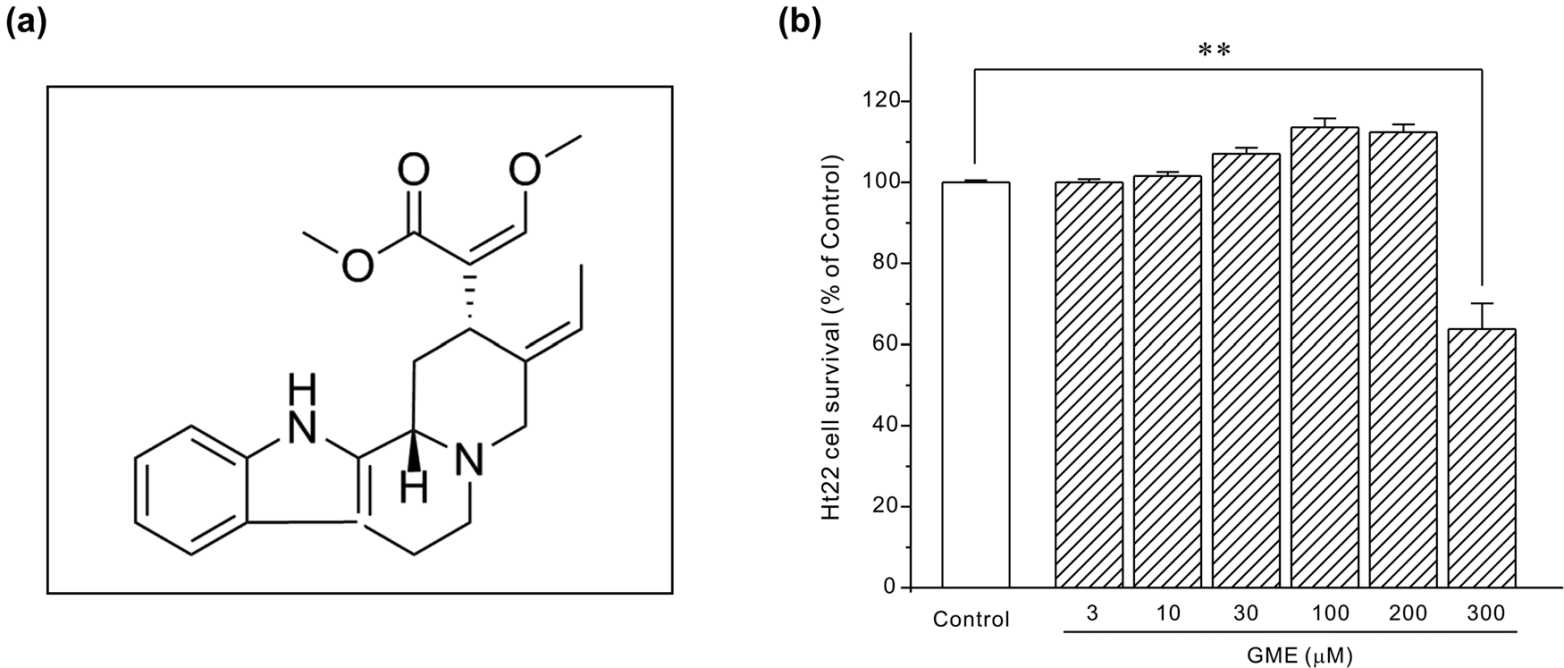
The possible toxic effect of GME on HT22 hippocampal neuronal cells: (a) chemical structure of GME and (b) HT22 neurons were incubated for 24 h by gradually increasing the dosages of GME (3–300 μM) or 0.1% DMSO (Control group), then examined with MTT assay. **, *p* < 0.01 versus Control group (*n* = 3).

Geissoschizine methyl ether (GME, Figure 1a) is an indole alkaloid originally isolated from the hooks of the Chinese medicine Gouteng (*Uncaria rhynchophylla*) and has been well characterized as a multifunctional compound *in vitro* and *in vivo*. For instance, GME effectively inhibited acetylcholinesterase with an IC_50_ of 3.7 μg/mL [13], suggesting that GME may enhance the amount of neurotransmitter acetylcholine in synaptic cleft to improve memory. GME has also been identified as a potent inhibitor of multiple neuronal channels [14] and as an agonist of serotonin 1A receptor [15]. Encouragingly, a research group analyzed the brain distributions of GME in rats injected intravenously with GME and found that in the 15 min-brain, GME signals were diffusely observed throughout the brain [16], indicating that GME could readily penetrate the blood brain barrier and has the potential to be developed as a neuroprotectant in treating neurodegenerative diseases. However, little is known about the neuroprotection and molecular mechanisms of GME against neurotoxins, H_2_O_2_ in particular. In this study, we reveal that GME protects HT22 neurons from H_2_O_2_-mediated damage possibly via inhibiting ERK pathway.

## 2 Materials and methods

GME was from Tauto Biotech (Shanghai, China). High-glucose Dulbecco’s modified Eagle’s medium (DMEM) and fetal bovine serum (FBS) were purchase from Gibco (Grand Island, NY, USA). Penicillin-Streptomycin solution (100×), Trypsin-EDTA solution was from Biosharp (Hefei, China). RIPA lysis buffer was from New Cell & Molecular Biotech (Suzhou, China). 2′,7′-Dichlorodihydrofluorescein diacetate (DCFH-DA) and 3-(4,5-dimethyl-2-thiazolyl)-2,5-diphenyl-2*H*-tetrazolium bromide (MTT) were from Sigma (St. Louis, MO, USA). Dimethyl sulfoxide (DMSO) was from Macklin (Shanghai, China). Primary antibodies for phospho-ERK (p-ERK), ERK (t-ERK), phospho-p38, p38, GAPDH, and β-actin were from Cell Signaling Technology (Danvers, MA, USA).

### 2.1 Culture of HT22 cells

HT22 mouse hippocampal neurons were from iCell Bioscience Inc. (Shanghai, China). Neurons were maintained in 100 mm petri dishes using DMEM that contained 10% FBS and penicillin–streptomycin (1×) in a 37°C CO_2_ incubator.

### 2.2 Measurement of neuronal survival

Neuronal viability was examined with MTT colorimetric method according to our earlier publication [17] based on the phenomenon that MTT could be transformed into formazan by succinic dehydrogenase in viable cells. HT22 cells were added into each well of 96-well microplates at a density of 1 × 10^4^ cells/well. On the next day, to test the toxicity of GME itself, cells were incubated with increasing dosages (3–300 μM) of GME for 24 h. To establish an oxidative stress model, HT22 cells were treated with increasing concentrations (50–500 μM) of H_2_O_2_ for 24 h. To test the protective effects of GME, cells were pre-incubated with GME (1–200 μM) for 2 h, and then treated with 350 μM H_2_O_2_ for 24 h. Thereafter, MTT solution was introduced into individual wells. The culture media were discarded after 3–4 h and the resulted formazan was suspended in DMSO. Absorbance values at 570 nm wavelength were immediately read in a microplate reader (SpectraMax i3x, Molecular Devices, USA).

### 2.3 Determination of intracellular ROS

The ROS amount was examined with a DCFH-DA probe based on the principle that DCFH-DA could be easily oxidized to fluorescent 2′,7′-dichlorofluorescein in the presence of generated intracellular ROS. Cells seeded in 96-well black plates were treated successively with 10 µM DCFH-DA for 1 h, 100 μM GME for 2 h, and 350 μM H_2_O_2_ for 15 min. Fluorescence changes were detected at excitation wavelength 485 nm and emission wavelength 535 nm.

### 2.4 Protein expression analysis

Protein expression was performed using Western blot as we reported [18,19]. In brief, HT22 cells in six-well plates were pre-treated with GME (3–100 μM) for 2 h, and then exposed to 350 μM H_2_O_2_ for different lengths of time. Culture media were removed and adherent cells were lysed in RIPA buffer supplemented with 1 mM phenylmethanesulfonyl fluoride. Cell extracts were transferred to Eppendorf tubes and subjected to centrifugation (4°C, 14,000×*g*, 10 min). Proteins in the supernatant were collected and subjected to the bicinchoninic acid assay. After denaturation at 100°C for 5 min, proteins (10–30 μg/lane) were loaded onto a 12% SDS-PAGE gel and transferred to PVDF membranes. The membranes were successively immersed into blocking solution for 1–2 h at room temperature, primary antibodies solution (1:1,000 dilution) overnight at 4°C, and respective secondary antibodies solution (1:2,000 dilution) for 1 h. Thereafter, membranes were developed with a Super ECL kit and exposed to X-ray films (Beyotime, Shanghai, China).

### 2.5 Molecular docking

The possible interaction and binding sites between GME and ERK2 protein were carried out using AutoDock 4.2.6 program. The initial structure was prepared using AutoDockTools 1.5.6, preserving the original charge of the protein and generating a pdbqt file for docking. The crystal structure of ERK2 was acquired from Protein Data Bank (PDB ID, 6OPK) and the 3D structure of GME was downloaded from the PubChem database. The active site of ERK2 was chosen as the binding pocket for docking. The number of grid points in the XYZ of grid box was set to 50 × 60 × 50, the grid spacing was 0.375 Å, the number of GA run was set to 100, rest of the parameters were set to default. Finally, the structure with the lowest docking energy was carried out with energy minimization.

### 2.6 Statistical analysis

All data are means ± SEM of independent experiments (*n* ≥ 3). Statistical analysis was performed using SPSS software. Multiple comparisons were carried out using one-way ANOVA followed by LSD’s *post-hoc* tests, *p* < 0.05 or less was taken as statistically significant.

## 3 Results

### 3.1 GME effectively promoted the survival of HT22 cells exposed to H_2_O_2_

First, the toxic effect of GME itself on HT22 cells was tested. As shown in Figure 1b, GME was non-toxic when its concentration did not exceed 200 μM and dosages less than or equal to 200 μM were then chosen for the subsequent neuroprotection assay.

**Figure 2:**
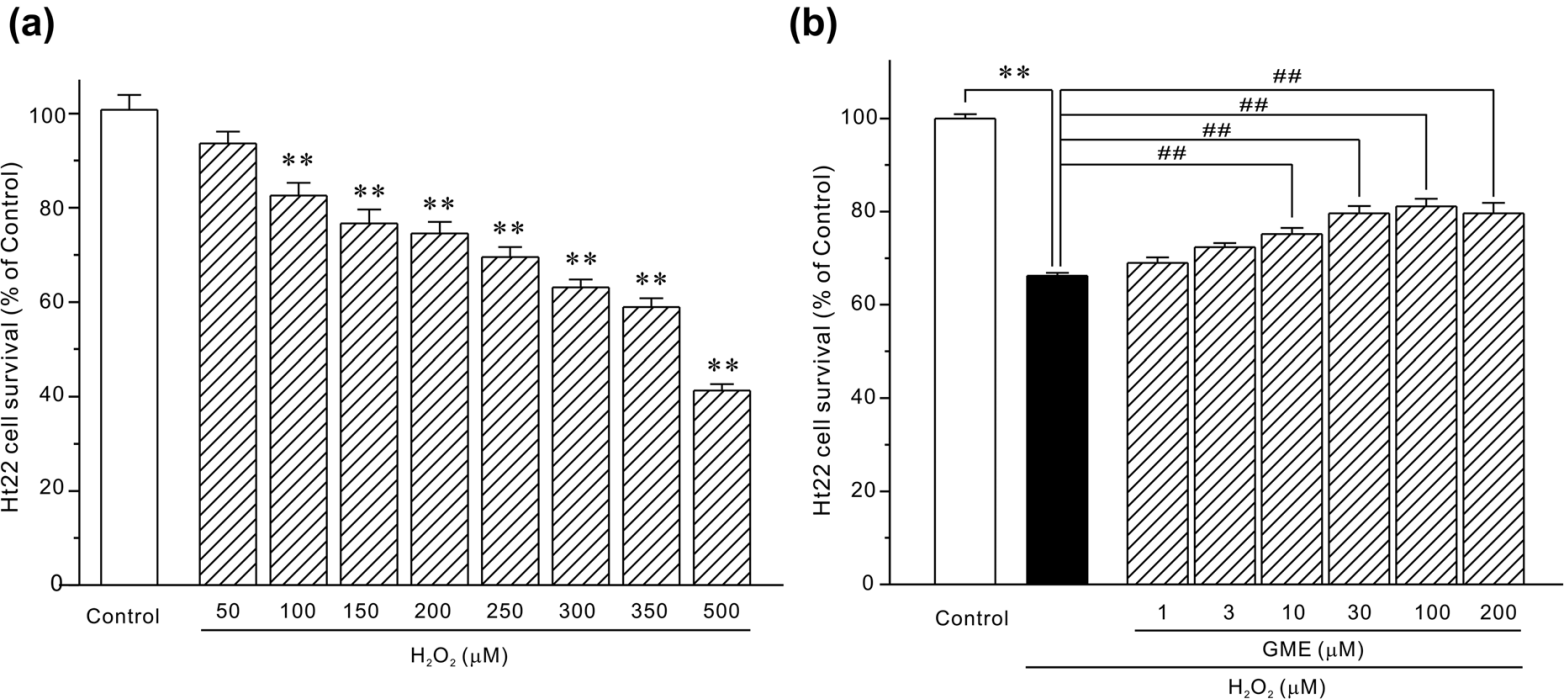
GME effectively protected against H_2_O_2_-induced injury in HT22 cells. (a) H_2_O_2_ dose-dependently decreased cell viability. Cells were insulted with H_2_O_2_ (50–500 μM) or dd H_2_O (an equal volume of H_2_O_2_, Control group) for 24 h and then tested by MTT assay. (b) GME significantly reversed cell survival inhibited by H_2_O_2_. Cells were successively treated with GME (1–200 μM) or 0.1% DMSO (Control group) for 2 h, H_2_O_2_ (350 μM) for 24 h, and tested with MTT assay. **, *p* < 0.01 versus Control group, ^##^, *p* < 0.01 versus H_2_O_2_ group (*n* = 4).

Second, an oxidative stress model associated with neurodegeneration was established by exposing HT22 cells to exogenous H_2_O_2_ solution. It is evident in Figure 2a that 24 h incubation with H_2_O_2_ (50–500 μM) dose dependently reduced the neuronal survival. Specifically, 350 μM H_2_O_2_ decreased cell viability to 58.47 ± 1.79% and this concentration of H_2_O_2_ was selected for the ideal inducer concentration. Two hours pre-treatment with GME (10–200 μM) promoted cell viability to 75.18 ± 1.33, 79.64 ± 1.52, 81.15 ± 1.62, and 79.64 ± 2.24%, respectively (Figure 2b).

### 3.2 GME significantly prevented abnormal morphological change challenged by H_2_O_2_ in HT22 cells

**Figure 3:**
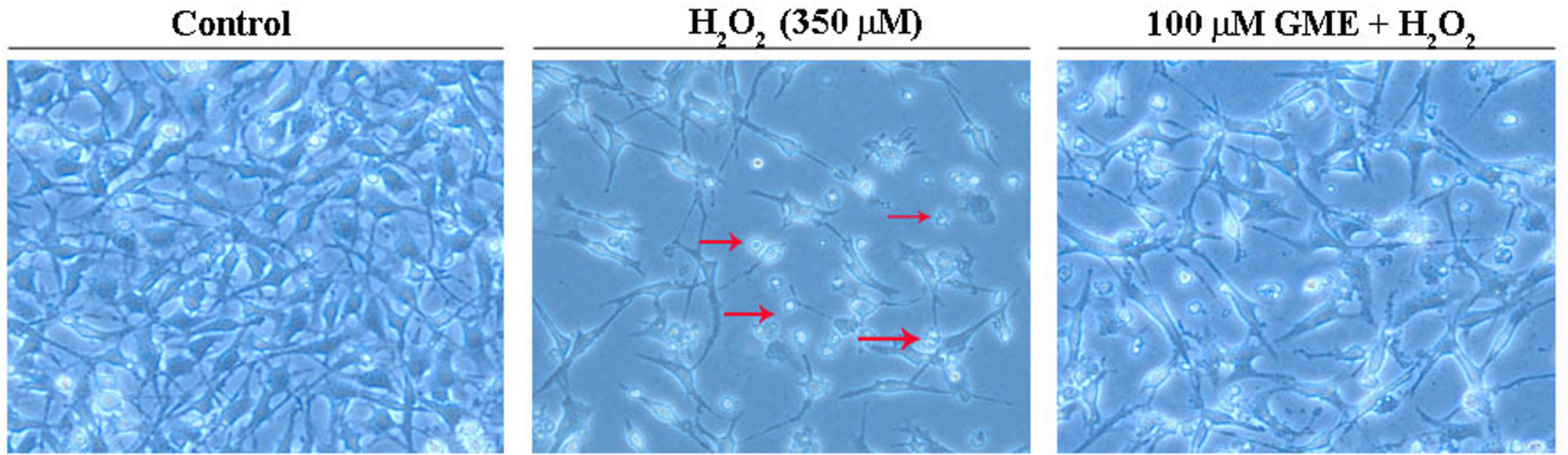
GME greatly blocked HT22 morphological changes challenged by H_2_O_2_. Neurons were successively treated with 100 μM GME or 0.05% DMSO (Control group) for 2 h, 350 μM H_2_O_2_ for 24 h, and then observed using a phase contrast microscope. Red arrows indicate damaged cells or intercellular connection (*n* = 3).

As observed in Figure 3, cells exposed to 350 μM H_2_O_2_ became too sparse, as compared to those in control group with high density. Moreover, these cells displayed abnormal cell characteristics, including round cell shape and damaged intercellular connection. Encouragingly, a remarkable increase in cell density and a decrease in the number of small round neurons were observed in the group of GME (100 μM) plus H_2_O_2_.

### 3.3 GME decreased intracellular ROS in HT22 cells

**Figure 4:**
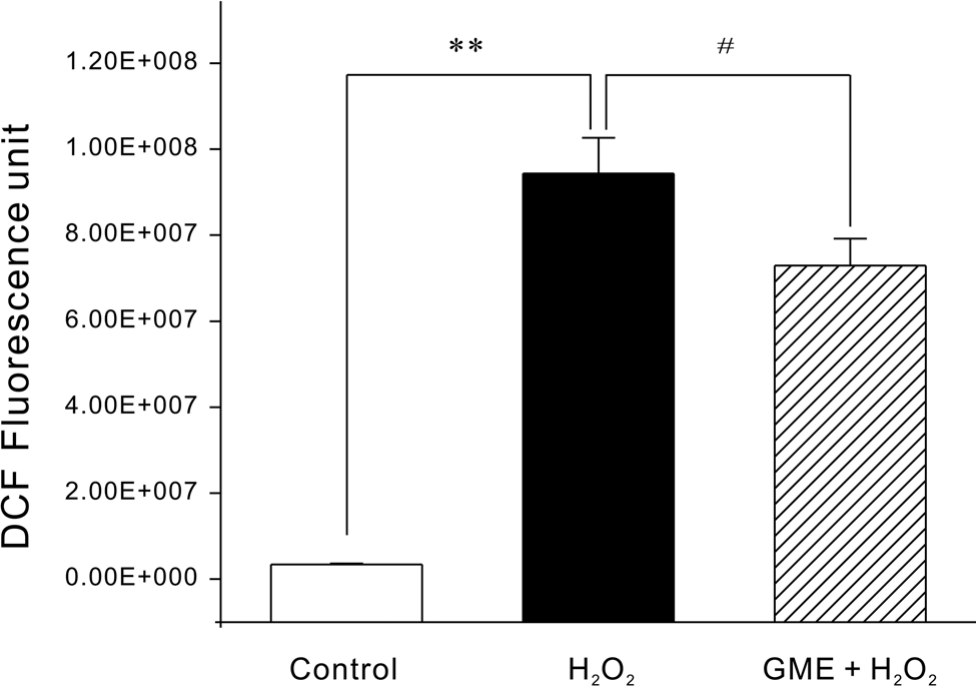
GME significantly inhibited the increase of intracellular ROS caused by H_2_O_2_. Neurons were successively treated with 10 µM DCFH-DA for 1 h, 100 μM GME or 0.05% DMSO (Control group) for 2 h, 350 μM H_2_O_2_ for 15 min. **, *p* < 0.01 versus Control group, ^#^, *p* < 0.05 versus H_2_O_2_ group (*n* = 3).

ROS resulted in oxidative stress and we thus tested the possibility that GME may inhibit intracellular ROS. As demonstrated in Figure 4, 350 μM H_2_O_2_, even 15 min after introduced into cells, caused a rapid and sharp increase in the intracellular ROS level as compared to control group. Pre-treatment of 2 h with 100 μM GME significantly decreased ROS production, equivalent to only approximately 77% of H_2_O_2_ group.

### 3.4 GME inhibited the increase of phospho-ERK, but not phospho-p38, protein expression caused by H_2_O_2_

**Figure 5:**
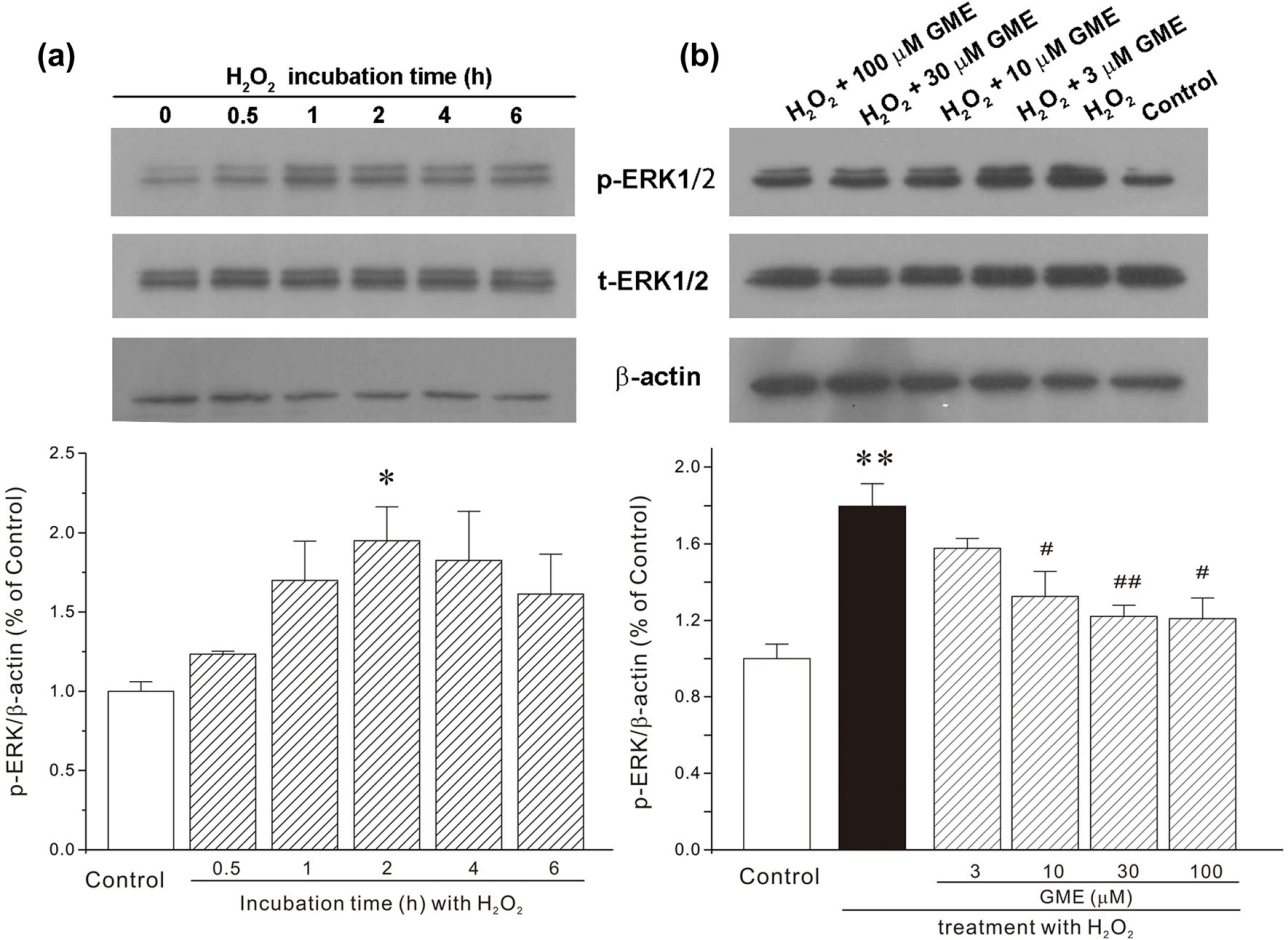
GME reversed the increase of p-ERK insulted by H_2_O_2_. (a) HT22 neurons were treated with 350 μM H_2_O_2_ or dd H_2_O (an equal volume of H_2_O_2_, Control group) for a short period of time (0.5–6 h) and protein analyses were performed with Western blot. (b) Neurons were successively treated with GME (3–100 μM) or 0.05% DMSO (Control group) for 2 h, 350 μM H_2_O_2_ for another 2 h. Protein was isolated and analyzed with Western blot using antibodies against p-ERK, t-ERK, and β-actin. *, *p* < 0.05 versus Control group, ^##^, *p* < 0.01 versus H_2_O_2_ group (*n* = 3).

**Figure 6:**
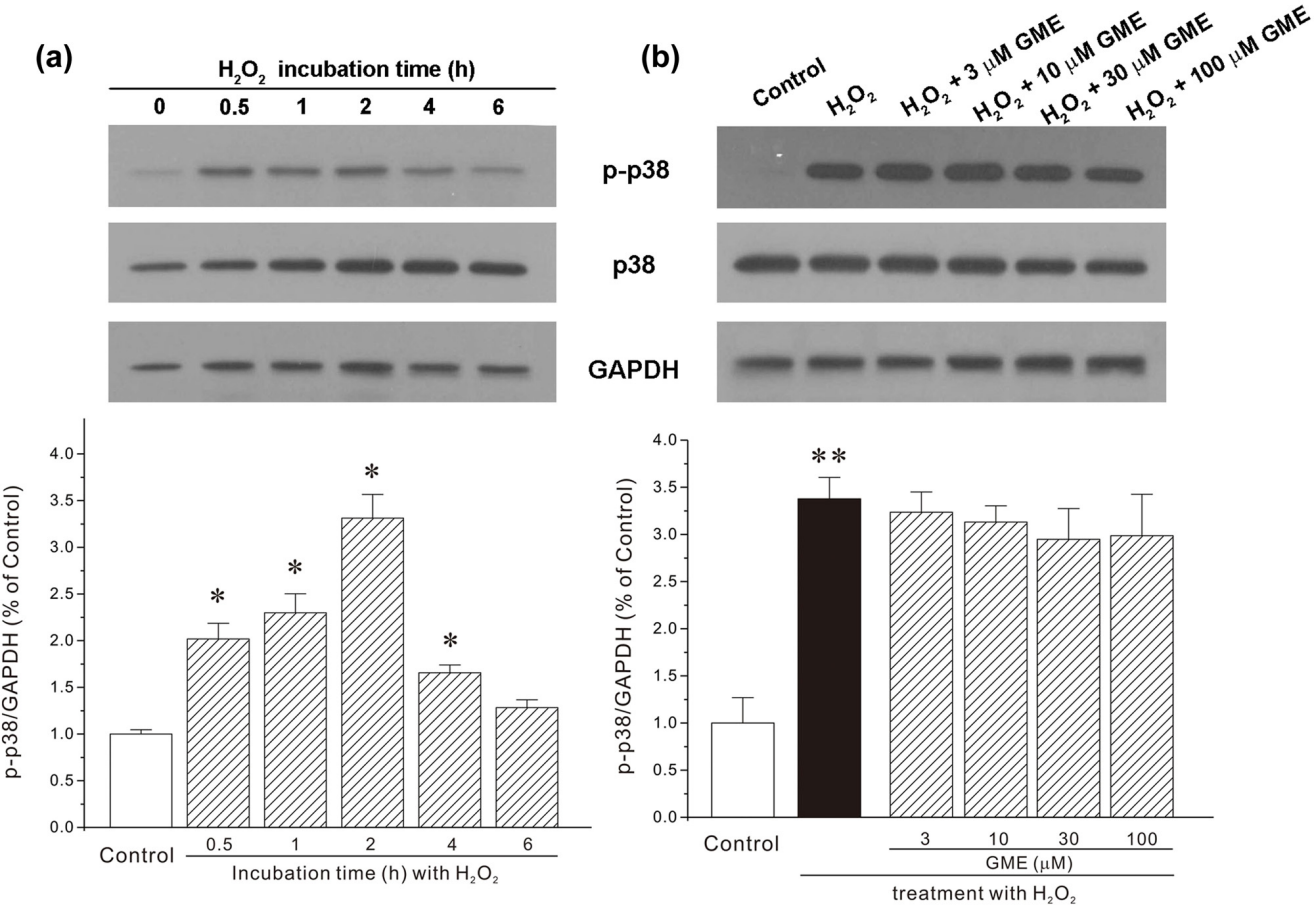
GME failed to restore the increase of p-p38 insulted by H_2_O_2_. (a) HT22 neurons were treated with H_2_O_2_ or dd H_2_O (an equal volume of H_2_O_2_, Control group) for a short period of time (0.5–6 h) and protein analyses were performed with Western blot. (b) Neurons were successively treated with GME (3–100 μM) or 0.05% DMSO (Control group) for 2 h, 350 μM H_2_O_2_ for another 2 h. Protein was isolated and analyzed with Western blot using antibodies against p-p38, p38, and GAPDH. *, *p* < 0.05 versus Control group, ^##^, *p* < 0.01 versus H_2_O_2_ group (*n* = 3).

MAPK pathway, extracellular signal-regulated kinase (ERK), and p38 in particular, were primarily evaluated in neuroprotection provided by GME. As shown in Figure 5, exposure of HT22 cell to 350 μM H_2_O_2_ evoked a significant increase in phospho-ERK (p-ERK) and phospho-p38 (p-p38), specifically, after H_2_O_2_ challenge for 2 h, both p-ERK and p-p38 reached a maximum level, which was 1.95 ± 0.21 and 3.31 ± 0.25-fold than that of the control level (Figures 5a and 6a). On the other hand, GME treatment gradually returned p-ERK protein to the normal level (Figure 5b). While, there was no changes in p-p38 protein expression among the groups of H_2_O_2_ and GME treatments (Figure 6b).

### 3.5 GME favorably bound to ERK2 protein via hydrophobic and hydrogen bonding interactions

**Figure 7:**
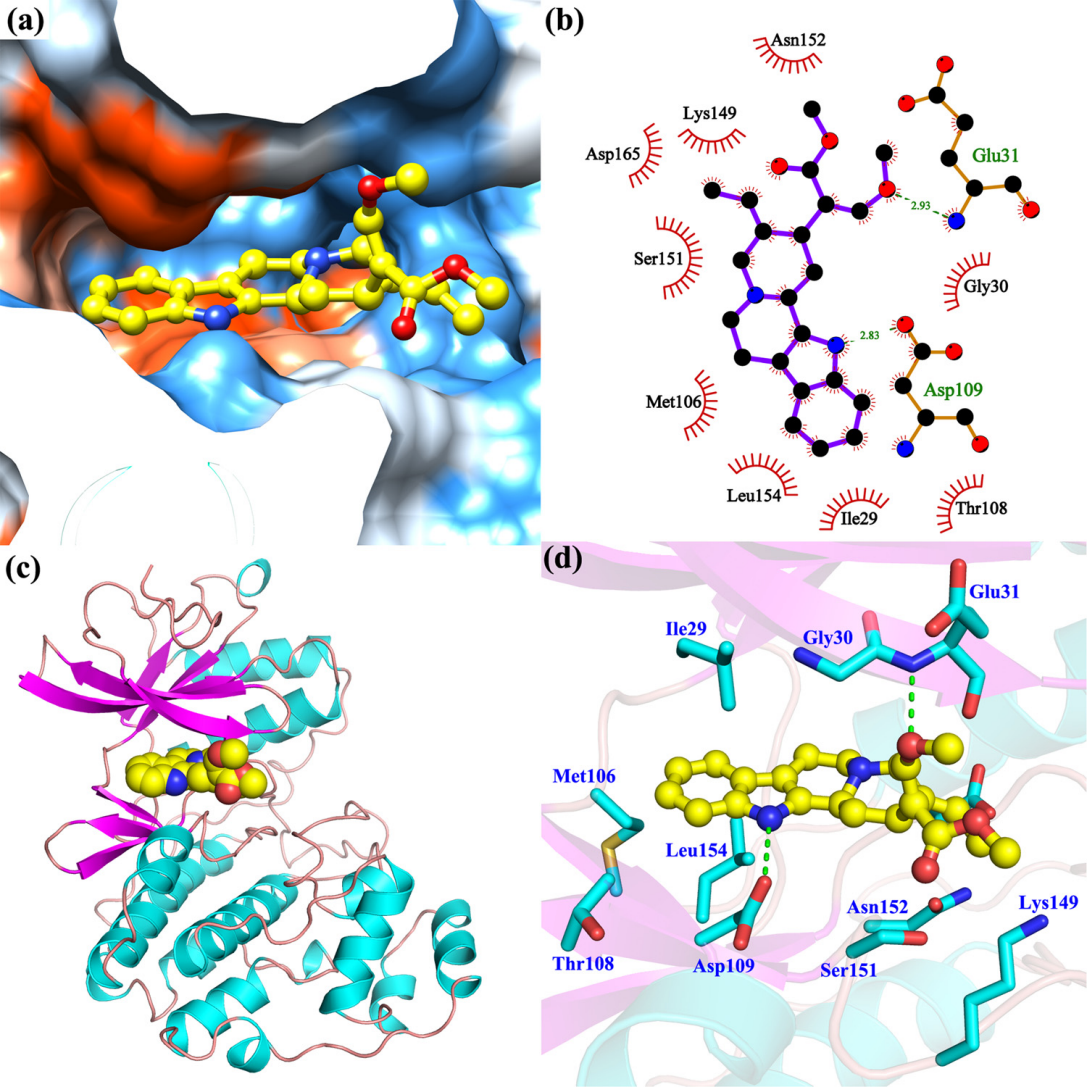
The possible binding mode of GME with ERK protein. (a) GME bound in the hydrophobic pocket of ERK (PDB ID, 6OPK). Blue color: hydrophilic domain, orange color: hydrophobic domain. (c) Binding position of GME in ERK protein. 2D (b) and 3D (d) ligand interaction diagram between GME and ERK. Dashed green lines indicated hydrogen bonds and red serrations represented hydrophobic interaction.

In our *in silico* molecular docking analysis, GME showed a moderate interaction with ERK2 (estimated binding energy = −5.60 kcal/mol). As shown in Figure 7, GME favorably bound to the active site of ERK2 protein. Specifically, GME was buried into a hydrophobic pocket consisting of Ile29, Gly30, Glu31, Met106, Thr108, Asp109, Lys149, Ser151, Asn152, Leu154, and Asp165. In addition, the aromatic ring of GME structure was stuck in the active cavity of ERK2 protein. In this binding position, the amino group of GME formed a hydrogen bond with Asp109, while the ester and ether parts in the GME structure formed another hydrogen bond with Glu31 near the hydrophilic groups outside the active site. Furthermore, there were also a large number of hydrophobic amino acids near the aromatic ring of GME, such as Ile29, Met106, Leu154, etc. These hydrophobic groups could further enhance the affinity between GME and ERK2 protein.

## 4 Discussion

Oxidative stress occurs when there is excess ROS that could not be eliminated immediately and are closely associated with the pathology of various neurodegenerative conditions [20,21]. Pharmaceutical augmentation of neuroprotective capacity is a potential means by which to prevent ROS-induced damage.

Chinese medicine and its associated medicinal herbs are huge treasure troves from which a variety of modern pharmaceuticals have been isolated and developed [22,23]. A good example includes huperzine A, an alkaloid derived from Chinese herb Qian Ceng Ta. Huperzine A was identified as an effective inhibitor of acetylcholinesterase [24], an enzyme responsible to hydrolyze neurotransmitter acetylcholine, and have thus been approved to treat Alzheimer’s disease in China. This discovery inspires us to pursue the naturally occurring neuroprotectant that could eliminate ROS from Chinese medicine herbs.

GME is a naturally occurring compound with less toxicity. In our study, we found that GME was non-toxic when its concentration did not exceed 200 μM. At a higher concentration (300 μM or above), GME became toxic. We speculate that GME over certain dose may cause inactivation of some key metabolic enzymes or decrease the activity of pro-survival transcription factor including myocyte enhancer factor-2 (MEF2) and cAMP responsive element binding protein. Moreover, the production of ROS and its associated oxidative stress process was induced by H_2_O_2_ in HT22 cells, a mouse-derived hippocampal neuronal cell line. Using this system, we found, for the first time, that GME provided effective neuroprotection against H_2_O_2_-induced cell death, a conclusion supported by the fact that GME promoted neuronal viability and decreased intracellular ROS. Collectedly, these findings encourage further pre-clinical investigation of GME in the potent application of neurodegeneration. Meanwhile, the cellular model and methodology established in this study may be used as a platform for screening neuroprotective compound with anti-oxidant ability in the modernization of Chinese medicine.

MAPKs, which are composed of ERK, p38, and c-Jun NH_2_-terminal kinase, function as important protein kinases in cell physiology [25]. In response to multiple extracellular and intracellular factors including neurotoxins or small chemical molecules, MAPKs are activated or inactivated, readily and instantly, to regulate diverse cellular activities including neuronal survival, differentiation, protection, and proliferation. ERK phosphorylation has been reported to be associated with neurodegenerative processes. Specifically, increased phospho-ERK level was usually observed in a variety of neurons and animals insulted by Aβ oligomers [26] and MPP^+^ [27], which represent neurotoxins that drive Alzheimer’s and Parkinson’ disease progression, respectively. Besides, an upregulation of ERK was involved in the activation of GSK3β, the progression of neurofibrillary degeneration as well as the hyperphosphorylation of tau [28,29]. Moreover, there is evidence suggesting that ERK signaling pathway regulated neuronal death by increasing p35 expression and by activating cyclin dependent kinase 5 (CDK5) [30]. As such, small molecules that could reduce ERK phosphorylation may provide effective neuroprotection against neurodegenerative diseases. In our model, an increase in protein expression of phosphorylated ERK, the subgroup of MAPK, was found in HT22 cells exposed to H_2_O_2_, an observation consistent with earlier studies [9,31]. GME dose-dependently reversed such increase of p-ERK1/2, indicating the involvement of ERK pathway in GME-mediated neuroprotection. Molecular docking simulation further revealed a possible binding mode that GME inhibited ERK protein, showing that GME2 favorably bound to ERK via multiple hydrophobic and hydrogen bond interactions. Meanwhile, there were no significant changes in p38 phosphorylation among groups, indicative of the absence of p38 signaling pathway in GME-provided neuroprotective mechanism. Nevertheless, we could not exclude any other possible important targets that H_2_O_2_ or GME may act on, such as MEF2, GSK3β, and CDK5.

**Figure 8:**
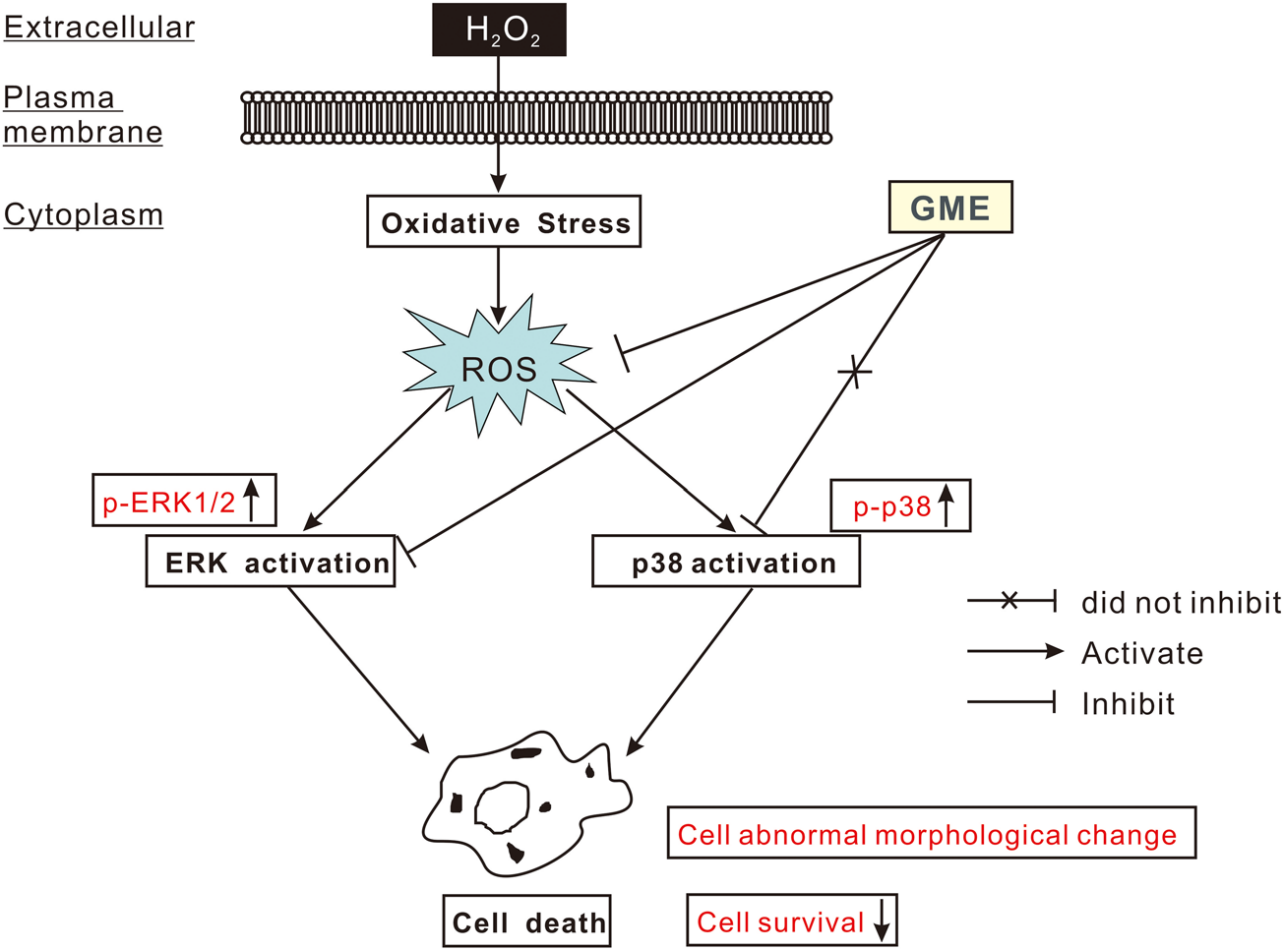
Schematic model revealing the molecular mechanisms by which GME protected against H_2_O_2_-induced toxicity. GME blocked the generation of excess ROS and its associated oxidative stress in HT22 cells, then decreased the protein level of p-ERK to inactivate ERK, finally prevented cell abnormal morphological change and promoted neuronal survival.

It should be noted that H_2_O_2_ induced various biochemical changes at different time points. For instance, it took just 15 min for H_2_O_2_ to trigger excess intracellular ROS, 2 h to induce ERK phosphorylation, 0.5–4 h to evoke p38 phosphorylation, while 24 h to decrease cell viability. This may be explained by the fact that ROS accumulation occupied an “upstream” position within the H_2_O_2_ toxicity cascade. Taken together, GME blocked the generation of excess ROS and its associated oxidative stress in HT22 cells, then decreased the protein level of p-ERK to inactivate ERK, finally prevented cell abnormal morphological change and promoted neuronal survival (Figure 8).

Recent studies have highlighted the multifaceted factors in the pathology of neurodegenerative conditions and the involvement of multi-target compounds in treating such devastating diseases [32,33]. These compounds are expected to synergistically hit different targets associated with neurodegenerative diseases in the brain to acquire better efficacy [34]. There is evidence suggesting that GME inhibited the activity of acetylcholinesterase [35], an enzyme responsible to hydrolyze neurotransmitter acetylcholine. In addition, GME was able to attenuate glutamate-induced excitotoxicity [36], a widely accepted hypothesis for Alzheimer’s disease. Thus, the neuroprotective effect and ERK inhibition ability described herein, together with the blockage of acetylcholinesterase enzyme activity and glutamate excitotoxicity reported earlier, may make GME as a promising anti-Alzheimer’s multi-target compound.

However, there are some limitations in the current study. First, GME-mediated inhibition of intracellular ROS should be confirmed by both DCFH-DA staining assay and fluorescence-activated cell sorting analysis. Second, since mitochondria are believed to be the main source of ROS in the neurons, it is reasonable to test the possibility that if GME may increase the mitochondrial membrane potential using TMRE staining. Third, though GME was found to inhibit the increase of p-ERK protein expression caused by H_2_O_2_ and bind to ERK via multiple hydrophobic and hydrogen bond interactions, the hypothesis that ERK activator (such as PAFC-16) or ERK SiRNA could abrogate GME-mediated neuroprotective effects needs to be verified to confirm the involvement of ERK pathway in GME-mediated neuroprotection. Such interesting topics will be carried out in our future projects.

## 5 Conclusion

We provide solid evidence that naturally occurring GME protects HT22 hippocampal neurons from H_2_O_2_-evoked neurotoxicity by inhibiting ERK pathway. Molecular docking simulation further reveals a possible binding mode that GME inhibits ERK protein, showing that GME2 favorably binds to ERK via multiple hydrophobic and hydrogen bond interactions. Our findings would encourage further *ex vivo* and *in vivo* pharmacological investigations of GME in treating oxidative stress-mediated neurological disorders.
